# Post-streptococcal acute glomerulonephritis complicated by gouty arthritis: a case report

**DOI:** 10.1186/s12969-015-0019-7

**Published:** 2015-06-17

**Authors:** Yasutaka Kuniyoshi, Azusa Kamura, Sumie Yasuda, Makoto Tashiro

**Affiliations:** Department of Pediatrics, Tsugaruhoken Medical CO-OP Kensei Hospital, 2-2-1 Noda, Hirosaki, Aomori 036-8511 Japan

**Keywords:** Gouty arthritis, Obesity, Post-streptococcal acute glomerulonephritis, Uric acid

## Abstract

Gouty arthritis is uncommon in childhood and adolescence. On the other hand, there has been no report of cases with development of gouty arthritis with post-streptococcal acute glomerulonephritis (PSAGN) in pediatric patients. Here we report the case of a mildly obese 12-year-old boy with PSAGN complicated by gouty arthritis of the left first metatarsophalangeal joint. On follow-up, it was confirmed that as serum C3 level returned to normal, urinary excretion of uric acid increased and serum uric acid level decreased, thereby resolving the burning pain of the left big toe. In this case, not only did renal insufficiency associate with PSAGN but also mild obesity may have led to hyperuricemia and gouty arthritis. In conclusion, clinicians should be aware that PSAGN may be complicated by gouty arthritis in obese pediatric patients.

## Background

Gout is a common medical problem [1, 2] that usually develops after middle age. Juvenile gouty arthritis, on the other hand, is a less common disease [3]. Hyperuricemia is known to be moderately to highly associated with renal dysfunction [4] and chronic glomerulonephritis in adults is often accompanied by hyperuricemia that results from decline in glomerular filtration rate (GFR) [5]. However, there have been no reports of cases with development of gouty arthritis with post-streptococcal acute glomerulonephritis (PSAGN) in pediatric patients. We describe the case of a mildly obese 12-year-old boy with PSAGN that was complicated by gouty arthritis of the left first metatarsophalangeal joint (MTP1). In obese patients, gouty arthritis may be induced by the renal insufficiency associated with PSAGN. Furthermore, in this paper, we report time-related changes in serum uric acid (UA) level, urinary excretion of UA (UA/creatinine ratio), and serum C3 level during follow-up of this patient.

## Case presentation

**Fig. 1:**
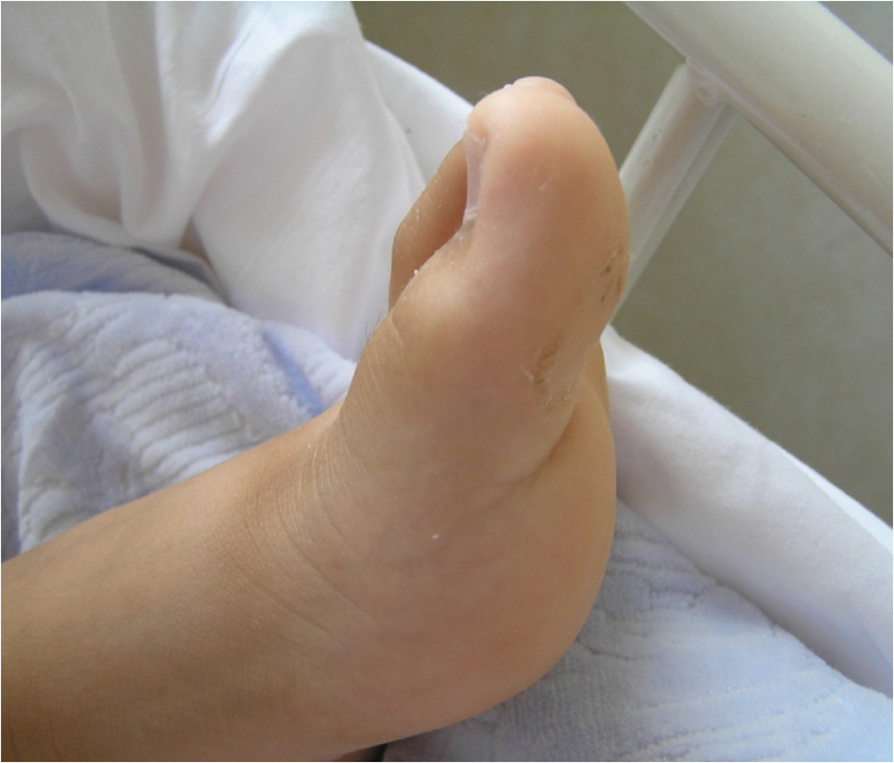
Gouty arthritis complicating PSAGN in a 12-year-old boy. The left MTP1 was swollen.

A 12-year-old Japanese boy with mild obesity but without significant medical history was admitted to our hospital because of hematuria, proteinuria, edema, and wet cough. He was a member of the sumo (Japanese-style wrestling) team in junior high school. Four days before admission, he took an antimicrobial agent for pharyngitis. After 2 days, he was noted to have abdominal pain, edema of the legs, and burning pain of the left big toe. His family did not have a history of hyperuricemia or gouty arthritis. On examination, his temperature was 36.8 °C, blood pressure was 160/92 mmHg, heart rate was 86 beats/min, respiratory rate was 22 breaths/min, oxygen saturation while breathing ambient air was 93 %; he weighed 84.9 kg (BMI 31 kg/m^2^) and was 165.1 cm tall. There was redness, swelling, and tenderness of the left MTP1 on light touch (Fig. 1). He was ambulatory but with conscious effort to avoid loading the left lower extremity with his body weight. Severe edema of both eyelids and lower legs was observed. The remainder of the examination was normal.

Laboratory examination revealed white blood cell count of 10900/μL, hemoglobin of 10.6 g/dL, hematocrit of 31.0 %, platelet count of 265000/μL, serum total protein level of 6.7 g/dL, albumin level of 3.1 g/dL, blood urea nitrogen (BUN) level of 30.8 mg/dL, serum creatinine level of 1.20 mg/dL, serum UA level of 10.5 mg/dL (reference range, <7.0 mg/dL), C-reactive protein level of 2.5 mg/dL (reference range, <3.0 mg/dL), C3 level of 25 mg/dL (reference range, 65–135 mg/dL), C4 level of 17 mg/dL (reference range, 13–35 mg/ dL), complement activities (CH50) level of 10U/mL (reference range, 29–48 U/mL), immunoglobulin A level of 174 mg/dL (reference range, 140–410 mg/ dL), and antistreptolysin O level of 628 IU/mL (reference range, <200 IU/mL). Dipstick urinalysis showed 2+ for occult blood and 3+ for protein; red blood cell casts and a few granular, hyaline, and erythrocyte casts were also observed. Group A beta-hemolytic streptococci were isolated from his throat culture. Computed tomography of the chest showed pulmonary edema and bilateral pleural effusion. Echocardiogram showed normal cardiac function and no valve disease.

**Fig. 2:**
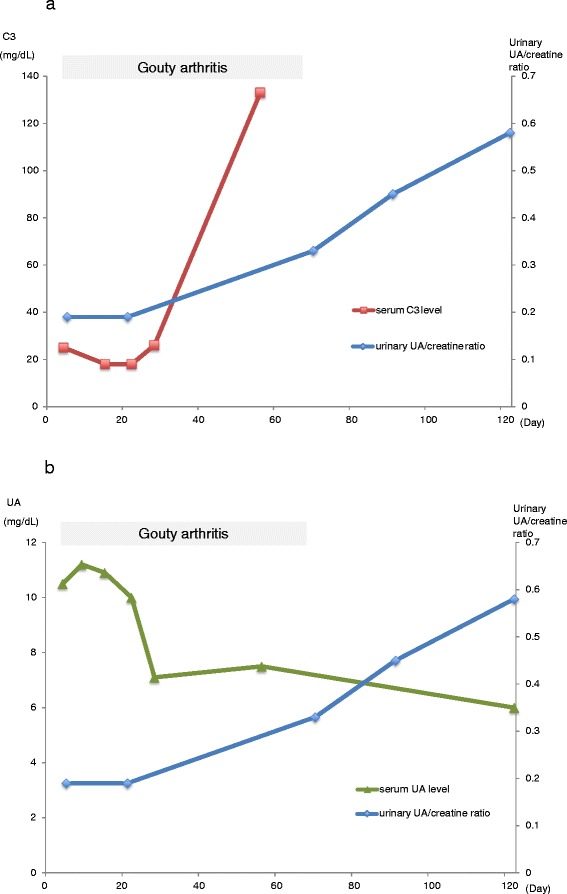
**a,b**: Laboratory values on follow-up. Time-related changes in serum UA level, urinary UA/creatinine ratio, and serum C3 level

The patient was treated with water and sodium restriction, oxygen administration at 2 L/min, intravenous furosemide, and oral amlodipine for hypertension and pulmonary edema; intravenous minocycline was also administered. He was initially treated for MTP1 gouty arthritic pain with oral acetaminophen. However, because of the persistence of pain, oral prednisolone (20 mg/day) was added on the 9th day. In addition, intravenous furosemide was discontinued because oxygen saturation improved and his weight decreased to 80.3 kg. On the 13th day, amlodipine was discontinued because blood pressure stabilized, with systolic pressure less than 120 mmHg. On the 26th day, he was discharged from the hospital; at this time, his body weight was 75.0 kg (BMI 27 kg/m^2^). The pain of the left MTP1 gradually improved and lasted for about 2 months after discharge. Figure 2 illustrates the changes in serum UA levels, UA/creatinine ratio, and serum C3 levels observed during the follow-up period.

## Conclusions

Juvenile gouty arthritis is a very rare disease [3]. The course of this patient provides an example of PSAGN that was complicated by gouty arthritis in an obese pediatric patient. In this case, gouty arthritis may have been induced by overproduction and under excretion of UA which were due to the renal insufficiency associated with PSAGN and mild obesity.

It is known that serum UA level is indirectly proportional to GFR in patients with chronic glomerulonephritis. This is due to impaired UA excretion from the kidney. Furthermore, it has been reported that hyperuricemia in IgA nephropathy was caused by both glomerular and tubulointerstitial damage [5]. We believe that hyperuricemia in PSAGN is caused by the same mechanism. Similar cases of juvenile gouty arthritis associated with mild renal insufficiency have been reported previously [6].

Our patient had probably been obese even before his clinical symptoms developed because his BMI was 27 kg/m^2^ after resolution of edema. Sumo players generally increase their body weight to gain an advantage. We hypothesize that mild obesity could also lead to hyperuricemia and gouty arthritis. Chen SY et al. reported that obesity could be responsible for the onset of hyperuricemia and juvenile gouty arthritis, as exemplified by their Taiwanese teenager patient [3]. Another case report [7] of a 15-year-old obese boy with a BMI of 47.4 kg/m^2^ has also been reported previously. The onset of gouty arthritis may depend on the balance in the production and excretion rate of UA [8]. Although our patient was mildly obese, we believe that this balance may have been disrupted because of the acute increase in serum UA level by PSAGN.

On follow-up, it was confirmed that as serum C3 level returned to normal, urinary excretion of UA increased and serum UA level decreased (Fig. 2-a, b). To the best of our knowledge, this is the first report on time-related changes in serum UA level, UA/creatinine, and serum C3 level in PSAGN. These findings suggest that the main mechanism of hyperuricemia in our patient was due to a decrease in UA excretion from the kidney. Current available evidence suggests that the major pathogenic mechanism responsible for this change is immune complex formation due to deposition of streptococcal nephritogenic antigens within the glomerulus [9]. Glomerular immune complexes activate the complement system and coagulation cascade, resulting to diffuse endocapillary proliferation and significant narrowing of the glomerular vessel lumen, which leads to a reduction in GFR. After the acute phase, glomerular immune complexes are cleared and the complement system is deactivated.

Loop diuretics may have contributed to the persistent pain on the MTP1 for almost two months. Diuretics, which reduce UA excretion by affecting ion exchanger proteins at the proximal tubule luminal membrane, are commonly withheld in patients with hyperuricemia [10, 11]. However, in the present case, in which the patient had pulmonary edema from fluid overload secondary to PSAGN, diuretics were necessary for fluid control. Therefore, in such cases, it may be necessary to monitor and watch out for the development of hyperuricemia.

This case report has some limitations. The diagnosis of post-streptococcal reactive arthritis (PSRA), acute rheumatic fever, and another etiology could not be excluded because synovial fluid analysis was not conducted. He was not diagnosed as having acute rheumatic fever according to the Jones criteria [12]. Simultaneous development of PSRA and PSAGN after subclinical streptococcal infection has been reported previously [13, 14]. The characteristics of PSRA as nonmigratory and persistent arthritis and tenosynovitis were applied to this case as well. Without synovial fluid analysis, the diagnosis of gouty arthritis was made according to the 1977 criteria for classification of acute arthritis from primary gout [15]. In reference to other clinical criteria reported by Janssens et al. [16], the probability of gout in our patient was more than 80 %, because of the presence of the following clinical features: male sex, joint redness, MTP1 involvement, and hyperuricemia [3].

In conclusion, clinicians must be aware that in obese pediatric patients, PSAGN may be complicated by gouty arthritis. In the future, we need to accumulate more cases and analyze the serum UA level of PSAGN patients with joint pain.

## Consent

Written informed consent was obtained from the patient’s parent for the publication of this report and accompanying images.

## Abbreviations

PSAGN: Post-streptococcal acute glomerulonephritis
GFR: Glomerular filtration rate
UA: Uric acid
BMI: Body-mass index
MTP1: First metatarsophalangeal jointl
PSRA: Post-streptococcal reactive arthritis
